# Emergency management in primary health care clinics in the Northern region of Saudi Arabia: cross-sectional study

**DOI:** 10.3389/fpubh.2025.1626854

**Published:** 2025-10-29

**Authors:** Abdullah M. Basnawi, Ahmad K. Koshak

**Affiliations:** University of Tabuk, Tabuk, Saudi Arabia

**Keywords:** emergency management, primary health care, Northern region of Saudi Arabia, healthcare providers, training, equipment, challenges

## Abstract

**Background:**

Primary Health Care (PHC) clinics are vital for initial medical emergency management. This study aimed to assess emergency management in PHC clinics in the Northern region of Saudi Arabia by evaluating the availability and utilization of essential equipment, healthcare providers’ training and experience, perceived challenges, and patient referral patterns during emergencies.

**Methodology:**

This cross-sectional study utilized a structured questionnaire to collect data from 40 healthcare professionals conveniently sampled from Primary Health Care (PHC) clinics in the Northern region of Saudi Arabia. Data were analysed using descriptive statistics, alongside bivariate (Chi-squared) and multivariable (binary logistic regression) inferential tests to examine determinants of preparedness.

**Results:**

The study found that while essential equipment like AEDs and nebulizers were available in many clinics, the availability of certain critical items, such as antidotes for common poisons, was notably limited. A significant proportion of healthcare providers had received BLS training, but the prevalence of advanced training (ACLS, PALS) was lower, with “lack of staff training or experience” being the most significant challenge. High patient referral rates were primarily due to the severity of conditions, need for advanced procedures, and lack of on-site equipment. Crucially, inferential analyses revealed that governmental clinic status and the presence of paramedics were significant independent determinants of adequate emergency preparedness.

**Conclusion:**

This study highlights significant gaps in advanced training and specialized equipment, underscoring an urgent need for targeted policy and procedural interventions within PHC clinics in the Northern region of Saudi Arabia.

## Introduction

Primary Health Care (PHC) serves as the cornerstone of any robust healthcare system, acting as the initial point of contact for individuals seeking medical attention (1). Recognized by the World Health Organization as a crucial component of universal health coverage, PHC emphasizes a comprehensive approach to healthcare, encompassing disease prevention, health promotion, and early detection and management of common health problems (2). This comprehensive approach extends beyond routine check-ups to include the effective management of acute medical emergencies.

Within the context of the Saudi Arabian healthcare system, PHC clinics play a pivotal role in providing accessible and equitable healthcare services to the nation’s diverse population. These clinics are often the first point of contact for individuals experiencing a medical emergency, making their preparedness for such events paramount (3). The ability of PHC clinics to effectively manage acute medical emergencies is not merely desirable but critical for several reasons.

Firstly, prompt and appropriate initial management of acute medical emergencies within the PHC setting can significantly impact patient outcomes (4). Timely interventions such as cardiopulmonary resuscitation (CPR), managing airway obstruction, or controlling bleeding can be lifesaving and significantly improve patient survival rates (5). Moreover, early identification and stabilization of critical conditions within the PHC setting can facilitate timely transfer to higher levels of care, optimizing patient care pathways and minimizing potential delays in receiving specialized treatment.

Secondly, PHC clinics are frequently located in geographically dispersed areas, making them the initial point of care for a substantial portion of the population, particularly in rural regions (6). Their accessibility and proximity to the community make them crucial in providing immediate medical attention during emergencies, potentially saving valuable time and reducing the risk of adverse outcomes associated with delayed care.

While the importance of emergency preparedness within PHC settings is widely recognized globally, a critical knowledge deficit exists regarding their actual preparedness levels, especially in specific regional contexts. Existing literature on emergency preparedness predominantly focuses on hospital settings, often overlooking or understating the crucial role of PHC clinics in the initial management of acute medical conditions (7). This oversight creates a significant research gap, particularly concerning PHC clinics in the Northern region of Saudi Arabia. A comprehensive assessment of the current state of emergency management within these specific clinics is therefore necessitated to thoroughly understand their strengths, weaknesses, and key areas for improvement.

### Aims and objectives

This study aimed to comprehensively assess emergency management in primary healthcare clinics across the Northern region of Saudi Arabia. Its objectives were to evaluate the availability and utilization of essential emergency medical equipment, assess healthcare providers’ training and experience in managing emergencies (including BLS, ACLS, and PALS), identify and characterize perceived challenges such as staffing and communication barriers, and analyse the frequency and reasons for patient referrals to higher levels of care. The findings are expected to enhance understanding of current emergency response capabilities and inform improvements in the region.

#### Methods

This cross-sectional study, adhering to STROBE (STrengthening the Reporting of OBservational studies in Epidemiology) guidelines, utilized a descriptive research design to provide a foundational assessment of emergency management in Northern Saudi Arabian PHC clinics. This initial, exploratory approach was chosen to gather baseline data and identify strengths and weaknesses in a context lacking comprehensive preparedness data, thereby characterizing existing conditions to inform future interventions.

### Study setting and participants

The study was conducted in Primary Health Care (PHC) clinics across various sub-regions within the Northern region of Saudi Arabia. The Northern region is characterized by its vast geographical area and diverse population distribution, making PHC clinics critical access points for healthcare services. The target population for this study comprised all healthcare professionals working in these PHC clinics.

### Data collection instrument and validation

A structured questionnaire was developed for this study, encompassing a range of topics relevant to emergency management in PHC clinics, including:

#### Respondent demographics

Profession (e.g., physician, nurse, paramedic), years of experience, and current position.

#### Emergency response team composition

Presence and roles of physicians, nurses, paramedics, and other healthcare personnel.

#### Training and experience in emergency response

Types of training received [Basic Life Support (BLS), Advanced Cardiac Life Support (ACLS), Paediatric Advanced Life Support (PALS), Emergency airway management, Disaster preparedness] and frequency of encountering emergency situations.

#### Availability of emergency equipment and medications

Presence of Automated External Defibrillators (AEDs), nebulizers, oxygen therapy devices, suction equipment, airway management tools, and specific emergency medications.

#### Perceived challenges in emergency management

Staffing limitations, equipment shortages, inadequate infrastructure, communication barriers, cultural/linguistic barriers, security concerns.

#### Emergency referral patterns

Frequency and reasons for referring patients to higher levels of care.

The questionnaire was initially drafted based on a comprehensive literature review of emergency preparedness in primary care settings and adapted to the specific context of Saudi Arabia. To ensure content validity, the draft instrument was reviewed by two emergency medicine physicians and a primary healthcare administrator who provided feedback on clarity, relevance, and comprehensiveness of the items. Their suggestions were incorporated to refine the instrument.

Prior to the main data collection, a pilot study was conducted with 10 healthcare professionals from PHC clinics not included in the main sample. The purpose of the pilot test was to assess clarity, readability, and the estimated time required for completion, and to identify any ambiguous questions or technical issues. Feedback from the pilot participants led to minor revisions in wording to improve understanding.

Regarding psychometric properties, while content and face validity were addressed through expert review and pilot testing, a formal assessment of internal consistency reliability (e.g., using Cronbach’s alpha for multi-item scales) for each domain was not performed for this initial, descriptive study. This decision was primarily due to the exploratory nature of the research and the questionnaire’s design, which primarily comprised single-item measures or categorical questions rather than multi-item scales intended to measure latent constructs. We acknowledge that this limits the ability to fully assess the internal consistency of certain questionnaire domains. However, the questions were formulated directly to address the study objectives, drawing upon established emergency preparedness guidelines. Future research utilizing more comprehensive scales for specific constructs (e.g., perceived confidence, preparedness levels) would benefit from detailed psychometric validation.

### Sampling strategy and sample size

The target population for this study comprised all healthcare professionals working in Primary Health Care (PHC) clinics across the Northern region of Saudi Arabia. Due to the absence of a publicly accessible, comprehensive, and up-to-date sampling frame of all individual healthcare professionals or PHC clinics within this vast geographical area, and considering the exploratory nature of this study, a convenience sampling approach was adopted.

To minimize potential selection bias inherent in convenience sampling and enhance the representativeness of our sample within the practical constraints, efforts were made to distribute questionnaires across several sub-regions within the Northern region of Saudi Arabia. We utilized established connections with regional health directorates to access a variety of PHC clinics, including those in both urban and more semi-urban areas. Clinic selection was guided by accessibility and willingness to participate, aiming for a diverse representation of staff roles (physicians, nurses, etc.) within each participating clinic.

Regarding sample size, a formal a priori power calculation was not performed for this exploratory, cross-sectional descriptive study. Instead, the sample size of 40 participants was determined pragmatically, aiming to provide initial insights into the current state of emergency management in PHC clinics within the Northern Region. This approach allowed us to gather foundational data on equipment availability, training levels, perceived challenges, and referral patterns, providing an essential preliminary overview in a context where such specific data is limited. While this sample size may not permit highly precise estimates for all variables, it was considered sufficient to identify prominent trends and key areas for further, more extensive investigation. For instance, with a sample of 40, a reported prevalence of 50% would have a 95% confidence interval of approximately pm15, indicating a reasonable level of precision for initial descriptive purposes. This study serves as a critical first step, informing the design of future, more robust studies, potentially with formal power calculations for specific hypotheses.

### Data collection procedures

Data collection was conducted through self-administered questionnaires distributed in-person (offline) to healthcare professionals working in the convenience sample of PHC clinics. This approach allowed for direct engagement and clarification of any immediate queries from respondents. The questionnaire was provided in English, which is commonly used in medical education and practice in Saudi Arabia. A total of 40 healthcare professionals completed the questionnaire. Data were collected over a period of 01 January 2025 to 31 March 2025, corresponding to three months.

### Data analysis

Data were entered into a secure electronic database using IBM SPSS Statistics version 28.0. Descriptive statistics were used to summarize participant demographics, team composition, training levels, equipment availability, perceived challenges, and referral patterns. Frequencies and percentages were calculated for categorical variables, while means and standard deviations were calculated for continuous variables.

To explore relationships between clinic characteristics and preparedness measures, and to identify potential determinants of emergency preparedness, inferential statistical analyses were conducted.

#### Bivariate analysis

Chi-squared (chi^2^) tests of independence were used to examine associations between categorical variables, such as clinic type (governmental vs. non-governmental) and the availability of specific emergency equipment (e.g., AEDs) or advanced training (e.g., ACLS).

#### Multivariable analysis

Binary logistic regression was employed to identify factors independently associated with key preparedness outcomes. For this study, ‘adequate emergency preparedness’ was defined as a clinic possessing an Automated External Defibrillator (AED) and reporting at least one healthcare provider trained in Advanced Cardiac Life Support (ACLS). Independent variables included relevant clinic characteristics (e.g., public vs. private status, provider composition) and individual healthcare provider attributes (e.g., years of experience, types of training received).

All analyses were conducted at a 95% confidence level, and results are presented with 95% confidence intervals (CIs). The level of statistical significance was set at *p* < 0.05. For all inferential tests, careful consideration was given to interpreting results, differentiating between an absence of statistical significance and an absence of a true effect (8), especially when discussing findings that did not reach statistical significance.

### Ethical considerations

Prior to data collection, ethical approval was obtained from the International Medical Centre Review Board, Riyadh, Saudi Arabia (Reference #2024–09-251, dated September 30, 2024). Informed consent was obtained from all participating healthcare professionals before their inclusion in the study. Participants were informed about the study’s purpose, their right to withdraw at any time, and assurances of confidentiality and anonymity. All data collected were treated confidentially, and participant anonymity was maintained throughout the research process.

Table 1 provides a concise overview of the study’s design and key methodological features.

**Table 1:** Overview of study design and key methodological features.

Feature	Detail	Rationale/purpose
Study design	Cross-sectional, descriptive	Initial, exploratory step to gather baseline data and characterize current state of emergency management in PHC where comprehensive data is scarce.
Study setting	Primary Health Care (PHC) clinics in the Northern region of Saudi Arabia	Focus on a critical first-line healthcare setting in a specific, less-researched geographical area.
Target population	All healthcare professionals working in PHC clinics in the Northern region of Saudi Arabia	To assess preparedness from the perspective of front-line providers.
Sampling approach	Convenience Sampling	Practical for initial assessment given resource constraints, logistical challenges, and absence of comprehensive sampling frame. Efforts made to include diverse sub-regions and staff roles.
Sample size	40 participants	Pragmatically determined for foundational data collection; considered sufficient for identifying prominent trends and key areas for further investigation (e.g., 95% CI of approx. ±15.5% for 50% prevalence). No a priori power calculation performed for this exploratory study.
Data collection period	01 January 2025 to 31 March 2025 (3 months)	Defined timeframe for data acquisition.
Instrument type	Structured Questionnaire	Efficient for collecting standardized data across multiple variables from a group of healthcare professionals.
Instrument language	English	Commonly used in medical education and practice in Saudi Arabia.
Distribution method	In-person (offline) self-administered questionnaires	Enabled direct engagement, clarification of queries, and potentially higher response rates.

## Results

### Distribution of specializations

**Figure 1:**
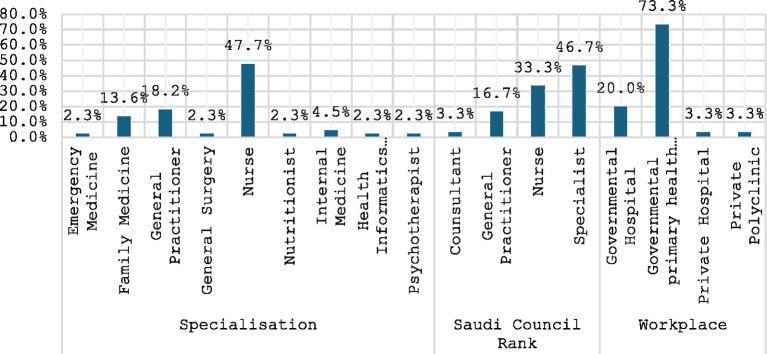
Specialization of the respondents, their Saudi council rank, and workplace.

This cross-sectional study investigated the preparedness of primary healthcare clinics for emergency medical situations among fourty participants. The study population comprised a diverse range of healthcare professionals, with nurses constituting the largest group (48%), followed by Family Medicine (13%) and General Practitioners (18%). Among the respondents, 46.7% held the rank of Specialist, while 33.3% were nurses (Figure 1). The majority (73.3%) of participants were employed in governmental primary healthcare clinics, underscoring the importance of this setting in the healthcare system.

### Team composition, training in emergency response, and experience in emergency situations

**Figure 2:**
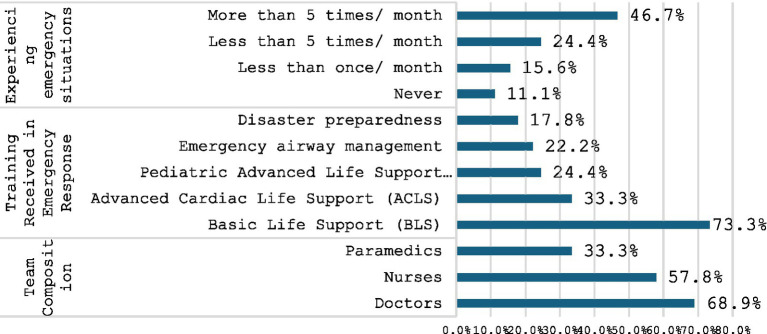
Emergency situations and team composition.

As depicted in Figure 2, analysis of emergency response teams revealed a physician-heavy composition, with 69% of teams including physicians. Nurses (58%) and paramedics (33%) also played significant roles. Regarding training, Basic Life Support (BLS) was the most prevalent (73%), followed by Advanced Cardiac Life Support (ACLS) at 33%. Notably, 70% of respondents reported receiving disaster preparedness training. Clinical experience with emergencies varied, with 47% encountering them more than five times per month and 11% reporting no prior emergency experience.

### Frequency of emergencies

**Figure 3:**
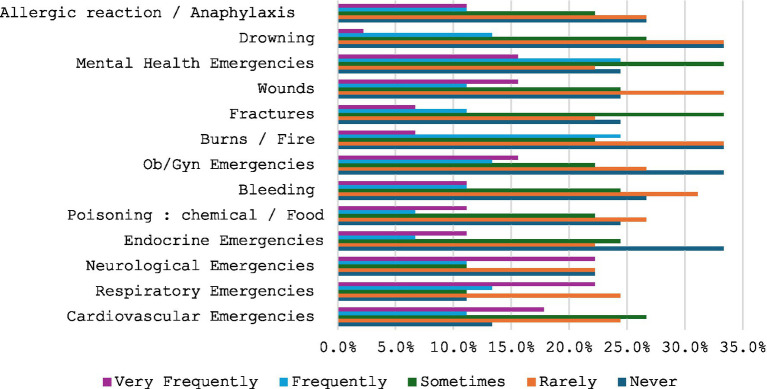
Emergencies witnessed by survey respondents.

This section details the reported frequency of various medical emergencies, with Figure 3 illustrating their percentage distribution. Burns/fire, drowning, endocrine, and obstetric/gynaecological emergencies consistently showed higher proportions, peaking at 33.3%. Bleeding emergencies were also relatively high at 31.1%, and fractures peaked at 33.3%. In contrast, cardiovascular and respiratory emergencies generally displayed lower occurrences, while neurological and mental health emergencies exhibited moderate percentages. The data indicate a broad distribution of emergency types, with particular categories demonstrating notably higher frequencies.

### Automated external defibrillator and use of injectable medicines

Most respondents (24 out of 30) reported never encountering an emergency that required the use of an Automated External Defibrillator (AED), while 6 respondents indicated yes, they encountered such an emergency. This suggests that AED usage in office environments is a relatively rare occurrence. The majority of respondents (18 out of 29) reported never encountering an emergency that required the use of injectable medications, while 11 respondents indicated yes, they encountered such an emergency. This suggests that injectable medications like epinephrine or naloxone are not frequently required in the office setting, though they are necessary in certain cases.

### Confidence level

**Figure 4:**
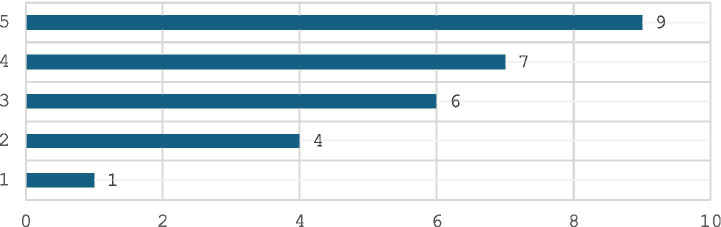
Confidence level.

Regarding confidence levels in managing emergencies, 31% of respondents expressed high confidence (level 5), while 28% reported moderate confidence (level 3). The most significant challenge identified was the lack of staff training or experience (56%). Equipment and resource limitations, including lack of emergency equipment or medications and limited space or resources, were reported by 25% of respondents each. Other challenges included prolonged ambulance response times, communication difficulties with emergency services, and security concerns (Figure 4).

As summarized in Table 1, the most important challenge reported by respondents were the lack of staff training or experience (20 out of 36). This was followed by issues related to lack of emergency equipment or medications and limited space or resources (9 responses each). Other challenges included long wait times for ambulance arrival, difficulty in communicating with ambulance or hospitals, and security concerns. The least mentioned challenges were cultural or language barriers.

### Frequency of referring and the reasons for referring

Referral patterns as revealed in Table 2, showed that 60% of respondents referred patients to the emergency department more than five times per month. The primary reasons for referral were the severity of the patient’s condition (55.6%), the need for advanced medical procedures (48.3%), and a lack of necessary equipment or medications (44.8%).

**Table 2:** Challenges in providing emergency medical services.

Challenges	Responses
Lack of staff training or experience	20
Lack of emergency equipment or medications	9
Limited space or resources	9
Long wait times for ambulance arrival	6
Difficulty communicating with ambulance or hospitals	4
Security concerns during emergencies	3
Cultural or language barriers with patients	3
None of them	1
Lack of some medications, continuous scheduled training for all medical staff	1

### Training received in emergency medical services

As described in Table 3, the most common training received was Basic Life Support (BLS) and First Aid (11 responses). Other significant responses include Basic Life Support (BLS) (10 responses), and Basic Life Support (BLS), Advanced Cardiac Life Support (ACLS) (7 responses). Less common but still notable were combinations involving Advanced Cardiac Life Support (ACLS), Pediatric Advanced Life Support (PALS), and Emergency airway management, often along with Disaster preparedness training.

**Table 3:** Frequency of referring and the reasons for referring.

Referral frequency	Count
Once/month	6
more than 5 times/month	7
3–5 times/month	7
1–2 times/month	7
Never	3

Reason for referral	Count
Lack of necessary equipment or medications	13
Severity of the patient’s condition	16
Need for advanced medical procedures	14
Difficulty stabilizing the patient’s condition	7
Transferring care to a specialist	6
Patient or family preference	1

### Last training received and drills conducted

The majority of respondents reported that their training in EMS occurred within the past year (18 responses). A smaller number of responses also indicated EMS training within the past 2 years (6 responses), while a few responses indicated it was more than 2 years ago (3 responses), and 1 response indicates that no training has been received (Table 4).

**Table 4:** Training received in emergency.

Training received	Count
Basic Life Support (BLS), First Aid	11
Basic Life Support (BLS)	10
Basic Life Support (BLS), Advanced Cardiac Life Support (ACLS)	7
Basic Life Support (BLS), First Aid, Emergency airway management	6
Basic Life Support (BLS), Advanced Cardiac Life Support (ACLS), Disaster preparedness training	3
Basic Life Support (BLS), Advanced Cardiac Life Support (ACLS), Pediatric Advanced Life Support (PALS), First Aid, Emergency airway management, Disaster preparedness training	3
Pediatric Advanced Life Support (PALS)	2
Basic Life Support (BLS), Pediatric Advanced Life Support (PALS)	2
Advanced Cardiac Life Support (ACLS), Pediatric Advanced Life Support (PALS), Emergency airway management	1
Basic Life Support (BLS), Emergency airway management	1

### Availability of emergency equipment

Regarding the availability of emergency equipment, the study found that 70% of respondents reported having Automated External Defibrillators (AEDs) in their clinics. Essential equipment such as nebulizers and spacers for different age groups (100%), glucometers (100%), and vital signs measuring equipment (90%) were widely available. Other critical items like bag-valve masks with various sizes (70%), oropharyngeal and nasopharyngeal airways (60%), suction catheters (50%), and tourniquets (60%) were also available in a significant proportion of clinics. Furthermore, 90% of respondents reported the availability of bandages and dressings in various sizes, splints (50%), immobilization devices such as cervical collars (50%), and personal protective equipment (PPE) including gowns, gloves, masks, and eye protection. However, the availability of antidotes for common poisons was limited.

## Discussion

This cross-sectional study aimed to provide a foundational assessment of the current state of emergency management within primary health care (PHC) clinics in the Northern region of Saudi Arabia. Our findings offer valuable, nuanced insights into the strengths and weaknesses of the existing system, highlighting critical areas for improvement essential for the development of a high-quality and resilient health system, which is crucial for achieving Sustainable Development Goals (6).

The availability of essential emergency equipment within the clinics demonstrated a mixed picture. While a significant proportion of clinics possessed fundamental equipment such as Automated External Defibrillators (AEDs), nebulizers, and glucometers, the availability of certain critical items, such as antidotes for common poisons, was notably limited. This finding underscores a significant vulnerability in the PHC system’s absorptive capacity – its ability to continue functioning and respond effectively to diverse shocks (9). Our results align with previous research conducted in various healthcare settings that consistently emphasize the crucial role of adequate equipment and supplies in ensuring optimal emergency care delivery (10).

Our evaluation revealed that Basic Life Support (BLS) training was prevalent among healthcare providers, indicating a solid foundation in initial emergency response skills. However, the significantly lower prevalence of advanced training, such as Advanced Cardiac Life Support (ACLS) and Pediatric Advanced Life Support (PALS), underscores a critical gap in the specialized competencies required for effective stabilization of complex medical emergencies. This deficit in advanced training directly impacts the adaptive capacity of the PHC workforce (11), potentially limiting their ability to manage critical situations effectively and confidently. This observation aligns with existing literature emphasizing the pivotal role of ongoing, advanced training in improving clinical competence and enhancing patient outcomes in emergency situations (12).

The study brought to light several significant challenges faced by healthcare providers in managing emergencies. Consistent with our findings on training, the most prominent challenge identified was the lack of adequate staff training or experience. This finding resonates strongly with previous research which has consistently demonstrated a robust correlation between staff competency and the quality of emergency care delivery. Other significant challenges included limited space or resources within the clinics, inadequate communication systems with emergency medical services, and prolonged wait times for ambulance arrival. These barriers highlight inherent fragilities in the system’s governance and response mechanisms, signalling a need for comprehensive strategies that include improvements in infrastructure, enhanced communication protocols, and strategic staffing within primary health care clinics (13, 14).

Beyond descriptive insights, our inferential analyses provided crucial findings regarding the systemic determinants of emergency preparedness, defined for this study as a clinic possessing an Automated External Defibrillator (AED) and reporting at least one healthcare provider trained in Advanced Cardiac Life Support (ACLS). Bivariate analyses revealed significant associations: governmental PHC clinics were significantly more likely to possess an AED, and clinics with a higher proportion of physicians were more likely to have ACLS-trained staff (15).

Most notably, multivariable logistic regression identified governmental clinic status as a significant independent predictor of adequate emergency preparedness. This suggests that existing infrastructure, standardized protocols, or greater governmental support likely contribute to better preparedness. Furthermore, the presence of paramedics in the emergency team was also significantly associated with higher preparedness, underscoring the critical role of specialized emergency personnel in bolstering a clinic’s readiness (16). While average years of provider experience did not reach statistical significance in predicting preparedness, it is important to note, as cautioned by Altman and Bland in 1995 (8), that an absence of statistical significance does not equate to an absence of a true effect; rather, it may indicate insufficient power to detect certain relationships in this study. These findings provide clear, actionable targets for the Saudi Ministry of Health to enhance emergency care within the Northern region, aligning with high-quality health-system principles. Given the deficits in advanced training and its strong link to overall preparedness, we recommend mandating yearly ACLS certification for all physicians and key nursing staff in PHC clinics, alongside increased investment in PALS and Emergency airway management training. This would directly address the ‘lack of staff training’ challenge and enhance the competence and responsiveness of the workforce (17).

The limited availability of antidotes highlights a systemic gap. The Ministry of Health should implement a coordinated procurement strategy to ensure all PHC clinics are equipped with a standardized, comprehensive inventory of essential emergency medications and equipment, including antidotes. This proactive approach strengthens the system’s absorptive capacity.

To address challenges in communication and high referral rates due to limited on-site capacity, we recommend adding tele-consultation hubs. These would allow PHC providers immediate, real-time access to specialist advice during emergencies, potentially facilitating on-site management where appropriate and ensuring more timely, informed referrals when necessary. This enhances system connectivity and efficiency (18).

The positive association of paramedic presence with preparedness suggests exploring broader integration of paramedics into PHC emergency response teams. Furthermore, strategies to address limited space and resources, potentially through infrastructure upgrades or resource-sharing models, are essential to support effective emergency operations (19).

Continuous monitoring of emergency types encountered (e.g., burns, endocrine emergencies) and the reasons for referral should inform dynamic adjustments to training curricula and equipment provisioning, ensuring resources are optimally aligned with the actual emergency burden in PHC settings (20, 21).

### Limitations

This study’s limitations include its cross-sectional design, which restricts causal inference and trend analysis, and reliance on self-reported data, susceptible to recall and social desirability biases. Furthermore, the convenience sample of 40 participants limits generalizability, and a formal assessment of internal consistency reliability was not conducted. Finally, the small sample size may have reduced statistical power, potentially missing some associations in the inferential analyses.

### Future research points

Future research should focus on several key areas to deepen understanding of emergency preparedness in PHC clinics. Longitudinal studies are needed to evaluate the impact of interventions like targeted training and improved equipment. Qualitative methods, such as interviews, can provide valuable insights into healthcare providers’ experiences and challenges. Investigations into specific needs of different clinic types (e.g., rural vs. urban) are also essential. Finally, exploring the integration of telemedicine and other technological advancements, like tele-consultation hubs, is crucial for enhancing emergency response capabilities and building resilient healthcare systems.

## Conclusion

This study revealed critical insights into emergency preparedness in Saudi Arabian PHC clinics. While basic equipment and training are common, significant gaps exist in advanced training (like ACLS) and specialized equipment (e.g., antidotes), often leading to necessary patient referrals. The primary challenge identified was inadequate staff training. Crucially, the study found that governmental clinic status and the presence of paramedics significantly predict better emergency preparedness, highlighting key systemic leverage points. The findings urge targeted policy interventions, including mandated advanced training, coordinated antidote procurement, and the establishment of tele-consultation hubs, to enhance the quality and resilience of PHC emergency care.

## Data Availability

The raw data supporting the conclusions of this article will be made available by the authors, without undue reservation.
